# Presentation of potential genes and deleterious variants associated with non-syndromic hearing loss: a computational approach

**DOI:** 10.5808/gi.21070

**Published:** 2022-03-31

**Authors:** Manisha Ray, Surya Narayan Rath, Saurav Sarkar, Mukund Namdev Sable

**Affiliations:** 1 Department of Pathology and Lab Medicine, All India Institute of Medical Sciences, Bhubaneswar, Odisha 751019, India; 2 Department of Bioinformatics, Odisha University of Agriculture and Technology, Bhubaneswar, Odisha 751003, India; 3 Department of ENT, All India Institute of Medical Sciences, Bhubaneswar, Odisha 751019, India

**Keywords:** computational approach, functional association network, miRNAs, non-syndromic hearing loss, single nucleotide polymorphism

## Abstract

Non-syndromic hearing loss (NSHL) is a common hereditary disorder. Both clinical and genetic heterogeneity has created many obstacles to understanding the causes of NSHL. The present study has attempted to ravel the genetic aetiology in NSHL progression and to screen out potential target genes using computational approaches. The reported NSHL target genes (2009‒2020) have been studied by analyzing different biochemical and signaling pathways, interpretation of their functional association network, and discovery of important regulatory interactions with three previously established miRNAs in the human inner ear as well as in NSHL such as miR-183, miR-182, and miR-96. This study has identified *SMAD4* and *SNAI2* as the most putative target genes of NSHL. But pathogenic and deleterious non-synonymous single nucleotide polymorphisms discovered within *SMAD4* is anticipated to have an impact on NSHL progression. Additionally, the identified deleterious variants in the functional domains of *SMAD4* added a supportive clue for further study. Thus, the identified deleterious variant i.e., rs377767367 (G491V) in *SMAD4* needs further clinical validation. The present outcomes would provide insights into the genetics of NSHL progression.

## Introduction

Hearing impairment is the most common neurosensory disorder [1] in humans. As of global statistics, approximately 360 million people have been suffered from hearing loss (HL) [2]. As a result, HL is reported as the fourth leading cause of disability in humans worldwide [3]. Meanwhile, congenital HL appears as the most prevalent chronic condition and defect in children [4,5]. The growing cases of hearing impairment in newborn are also reported by the universal newborn hearing screening board [5]. It is also identified that 70% of congenital HL accounts for non-syndromic hearing loss (NSHL) [6] whereas the rest 30% comes under syndromic [7]. As per literature, molecular and genetic aetiology is the prime cause of congenital HL. But, due to genetic and clinical heterogeneity in NSHL, the disease pathogenesis [6] is not clearly understood. Therefore, there is a subsequent delay in the discovery of disease pathways and identification of molecular targets since 1990 [8]. Again, complexity in the identification of potential target genes and their associated variants at an accurate genomic location has created lots of diagnostic complications in NSHL [9].

The association of several important genes such as *GJB2, GJB3, GJB6, SLC26A4, KCNQ4, DFNA5, SLC26A5, MYO1A, MYO7A, MYH15A, *and *CDH23* has been identified with respect to the progression of congenital NSHL. Among these, GJB2 is established as an imperative target of NSHL [9]. In addition, the successful adoption of genome-wide association studies in biomedical research has discovered the genetic architecture of novel variants and their disease associations [10,11] which encourages researchers to focus on the identification of a potential biomarker for NSHL.

Generally, identification of potential biomarkers through the integration of multiple complementary approaches [12] is better than using any single method. As of evidence, many studies have been reported using more than one in silico methods such as pathway enrichment and functional annotation or protein interaction (PPI) network to screen and/or discover potential target genes from a large scale gene pool in diseases like atherosclerosis, nasopharyngeal carcinoma, systemic sclerosis, Parkinson disease, papillary thyroid carcinoma, neurodegeneration, etc. [13-19]. So, to identify any putative target genes, different aspects including the study of different regulatory pathways, miRNA-based gene regulation, annotation of protein functional network, and variant analysis are need to be addressed. Particularly, the discovery of important biological pathways involved in signal transductions [20] has a great aid and value. In this connection, the study of functionally important protein-PPI provides a meaningful direction towards understanding the involvement of disease targets in different biological processes [20]. Similarly, the discovery of miRNAs-based gene regulatory networks in the post-transcriptional process has a certain importance in biomarker identification [21]. As proof, experimental studies have also shown the involvement of a few miRNAs (miR-182, miR-183, and miR-96) in human inner ear development [22-25]. In addition to this, detection of deleterious single nucleotide polymorphisms (SNPs) has also been implemented in biomarker identification to ascertain disease susceptible genes. Especially, non-synonymous SNPs in the coding region have a vital role to produce various damaging effects on protein structure, stability, charge, etc., which leads to functional dysfunction of several disease targets [26-28]. Therefore, the identification of non-synonymous SNPs within the proper coding architecture of disease targets is an important strategy of biomarker identification. The present study is focused on the discovery of potential NSHL targets and their functional variants using multiple silico complementary approaches.

## Methods

### Data mining

A systematic review of literature was carried out by following the guidelines of Preferred Reporting Items for Systematic Reviews and Meta-Analyses (PRISMA). The scientific articles on NSHL were searched in PubMed (https://pubmed.ncbi.nlm.nih.gov/), Science Direct (https://www.sciencedirect.com/), Cochrane Library (www.cochranelibrary.com/), and JSTOR (www.jstor.org/) databases using MeSH terminologies such as “Non syndromic hearing loss” (congenital non syndromic hearing loss* (MeSH), non-syndromic hearing loss* (MeSH), non-syndromic hearing loss (MeSH)) and “genetics” (gene* (MeSH), genetics* (MeSH)), AND “epidemiology & pathogenesis” (epidemiology* (MeSH), pathogenesis*(MeSH)). The articles were collected from the last decade up to December 2020. Following inclusion and exclusion criteria were followed to select the appropriate literature for reference.

### Inclusion criteria

The following criteria were followed for selecting the articles:

(1) Literatures providing information about target genes involved in NSHL.

(2) The articles published in the English language.

(3) The full-length article available from period 2009-2020.

All available full-length original research articles and case reports were included.

### Exclusion criteria

The rest of the literatures were excluded based on the following exclusion criteria:

(1) The review articles, abstracts, articles written in other languages, letters to the editor, short reports, and correspondences.

(2) The articles are based on syndromic HL and the adult population.

(3) Articles without any genetic information.

### Dataset preparation

The NSHL target genes were selected from shortlisted literatures and subjected for removal of duplicate genes. The unique NSHL target gene list was prepared for further analysis.

### Discovery of NSHL pathway

All of the selected genes were employed to discover their association in different biochemical and signaling pathways involved in the development and progression of NSHL using KEGG (Kyoto Encyclopedia of Genes and Genomes) Mapper web server (https://www.genome.jp/kegg/mapper.html).

### Functional network analysis

The pathway associated NSHL target proteins were analyzed using STRING (https://string-db.org/) web server in order to represent a functional network between them.

### Discovery of regulatory miRNAs-gene interaction in NSHL progression

Functionally associated target genes were subjected to miRTarBase (http://miRTarBase.cuhk.edu.cn/) web server to analyze their regulatory interaction with three previously reported miRNAs (miR-182, miR-183, and miR-96). The identified miRNA-gene interaction hubs were validated and graphically represented through miRNet (https://www.mirnet.ca/) tool.

### Variant analysis

Effective SNPs were predicted from NSHL target genes to identify their functional variants. SNPs were searched from dbSNP database (https://www.ncbi.nlm.nih.gov/snp/). Validation of SNPs were performed using SIFT (https://sift.bii.a-star.edu.sg/) [29], PredictSNP1 (https://loschmidt.chemi.muni.cz//predictsnp1/), and PredictSNP2 (https://loschmidt.chemi.muni.cz/predictsnp2/) algorithms [30].

**Fig. 1.**
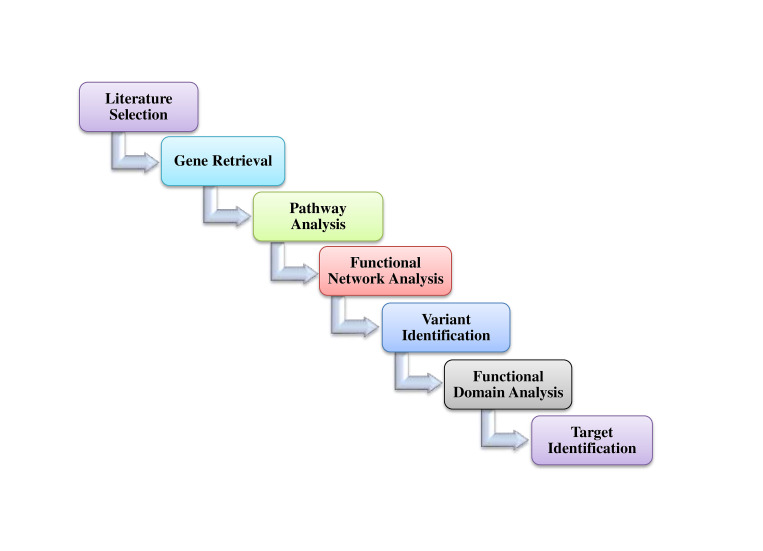
Schematic representation of complete methodology followed in the present study.

The schematic representation of overall procedures is depicted in Fig. 1.

## Results

### Selection of target genes

**Fig. 2.**
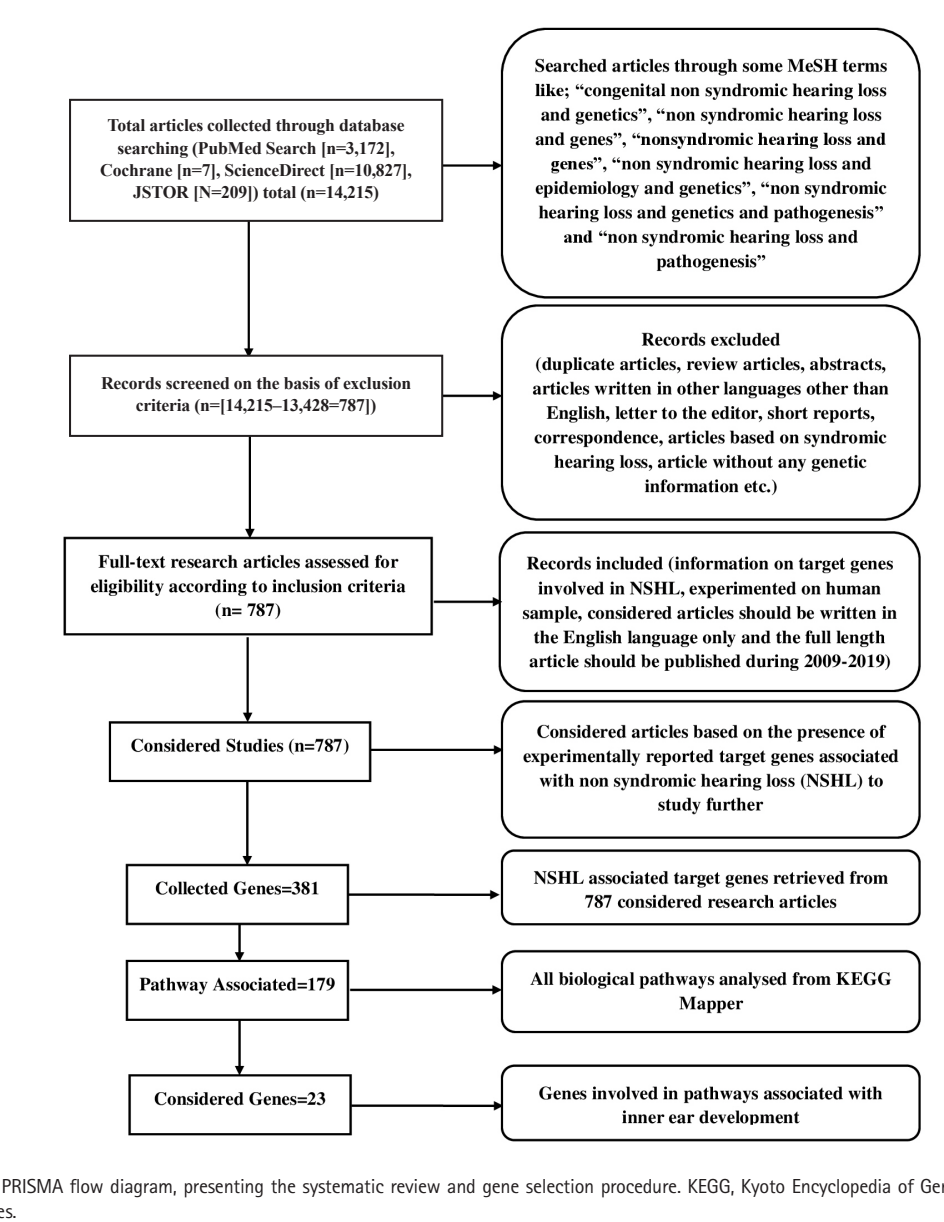
PRISMA flow diagram, presenting the systematic review and gene selection procedure. KEGG, Kyoto Encyclopedia of Genes and Genomes.

Total 14,215 numbers of articles were selected on the basis of PRISMA guidelines from different scientific repositories such as PubMed (3,172), JSTOR (209), Science Direct (10,827), and Cochrane (7). Among these, a total of 787 studies were satisfied with all of the inclusion criteria and considered for this study (Fig. 2), whereas the remaining studies were excluded as per the exclusion criteria manually. Further, a total of 2,707 NSHL target genes were collected from these 787 studies, out of which 422 genes were identified as unique. Again, from these 422 unique target genes, mitochondrial RNA (5), DNA (1), and microRNAs (7) were discarded, and the rest of 381 genes (Fig. 2) were considered as the final data set of NSHL targets.

### Analysis of NSHL target genes

**Fig. 3.**
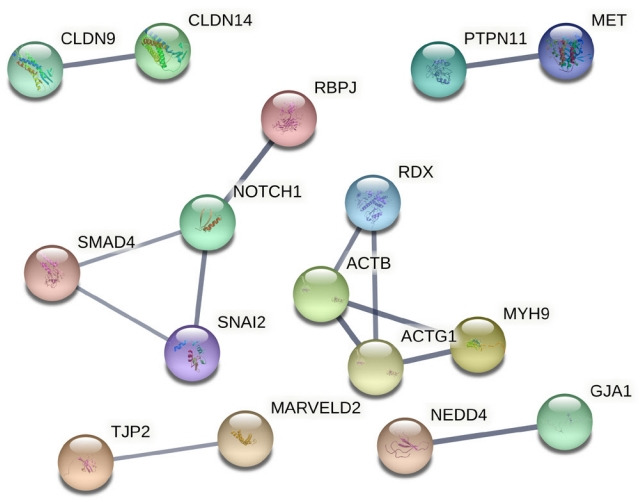
Functional association between non-syndromic hearing loss target genes were presented through protein-protein network at high confidence level. Edge thickness represents the confidence of association between nodes (proteins) within the network.

The association of 381 selected genes was searched against different biological pathways to discover potential NSHL targets. It was interpreted, a total of 23 genes (Fig. 2) are significantly involved in six different pathways such as Notch signaling pathway (hsa04330), Wnt signaling pathway (hsa04310), gap junction (hsa04540), tight junction (hsa04530), JAK-STAT signaling pathway (hsa04630), and adherens junction (hsa04520) which are associated with NSHL development (Table 1). Therefore, these 23 genes such as *NOTCH1, RBPJ, LRP5, SMAD4, RAF1, ADCY1, GJA1, *SNAI2*, ACTB, ACTG1, FGFR1, MET, TJP2, CLDN14, CLDN9, MARVELD2, MYH9, NEDD4, NF2, RDX, IFNLR1, IL13,* and *PTPN11* were anticipated as potential NSHL targets. Afterward, functional association of these 23 NSHL targets was studied using the STRING tool with the high level of confidence parameter (score 0.007). The resulting protein-protein network showed a strong interaction between 16 nodes (genes) (Fig. 3) with a significant PPI enrichment p-value of 0.00107. Particularly, strong functional interactions have been observed between six groups of targets i.e., *RBPJ, NOTCH1, SNAI2*, and *SMAD4; RDX, ACTB, ACTG1, and MYH9; CLDN9-CLDN14; TJP2-MARVELD2; PTPN11-MET; NEDD4-GJA1* in the resulted PPI network. Further, the involvement of these 16 NSHL target genes was also confirmed in a few hearing associated biological processes such as inner ear development, inner ear auditory receptor cell differentiation, homeostatic process, chemical homeostasis, signal transduction, regulation of response to external stimulus, related to hearing development and impairment (Table 2).

**Fig. 4.**
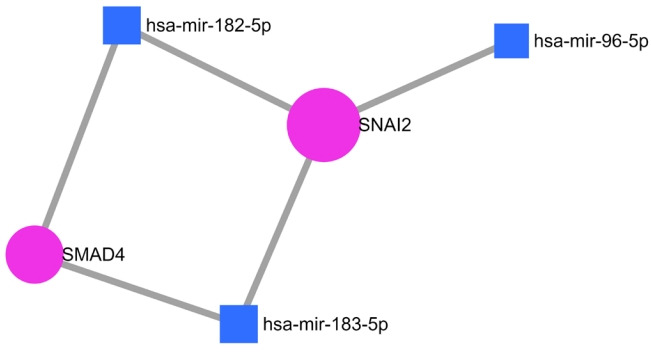
Strong regulatory network is identified between miRNAs established in development of inner ear signaling pathway and putative non-syndromic hearing loss (NSHL) targets. Blue color box, miRNAs; Purple color sphere, NSHL target genes.

Further study was performed to analyze the gene regulatory network between these 16 NSHL target genes with three important miRNAs such as miR-182, miR-183, and miR-96 which are significantly expressed in the human inner ear and associated with NSHL. Among these, the strongly validated interaction was found between the target genes *SMAD4* and *SNAI2* with miR-182 and miR-183, whereas miR-96 regulates only *SNAI2* (Fig. 4).

### Identification of deleterious variants in NSHL target genes

**Fig. 5.**
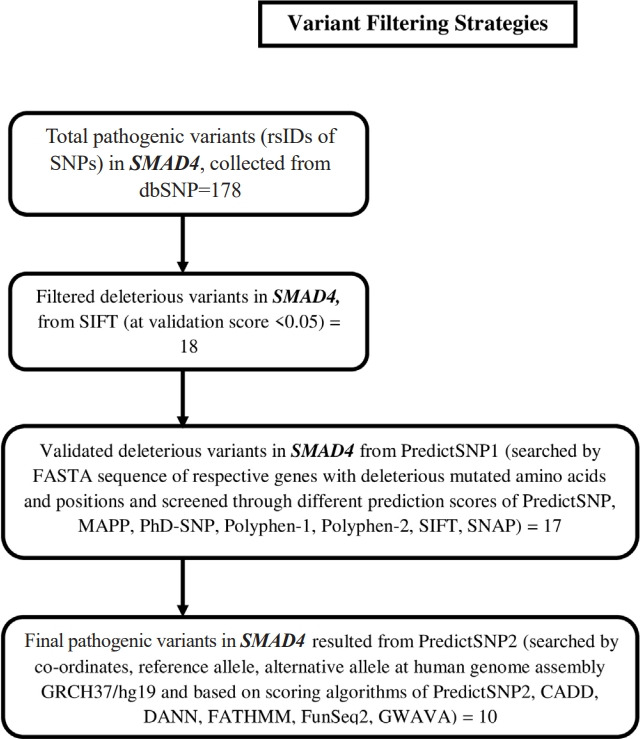
Variant filtering strategies are presented using flow diagram.

Non-synonymous SNPs (nsSNPs) were predicted for two important NSHL targets (*SMAD4*, *SNAI2*) confirmed from previous analysis. The prediction was resulted total 178 rsIDs of pathogenic variants for *SMAD4* gene with clinical significance from dbSNP (Supplementary Fig. 1). At the same time no pathogenic variant was obtained for *SNAI2* gene. Initial validation using SIFT algorithm (<0.05) was identified 18 rsIDs (out of 178 rsIDs) in *SMAD4* with deleterious effect (Supplementary Fig. 2). Afterwards, cross validation using PredictSNP1 was resulted 17 rsIDs in *SMAD4* (Supplementary Fig. 3) as deleterious variants. Finally, theses variants were further validated using PredictSNP2 algorithms which was resulted 13 numbers of deleterious nsSNPs (Table 3, Fig. 5) assigned with 10 rsIDs i.e., rs377767345 (G352E), rs121912581 (G352R), rs377767347 (R361H, R361L), rs377767348 (C363R), rs121912580 (G386D), rs377767367 (G395V, G491V), rs377767369 (W509R), rs377767371 (G510V), rs377767382 (L533P, L533R), rs377767381 (L533V).

**Fig. 6.**
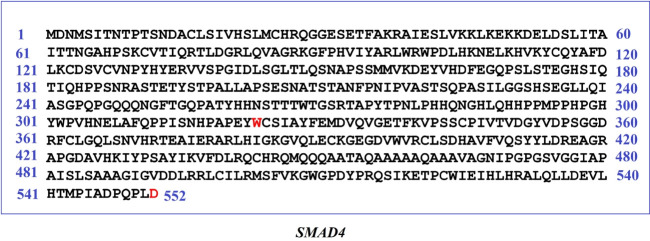
Predicted non-synonymous single nucleotide polymorphisms within the functional domain regions of putative non-syndromic hearing loss target (SMAD4) are highlighted.

Furthermore, functional domain regions of *SMAD4* were analyzed from the respective protein structure. Interestingly, all of these 13 deleterious variants (Table 3) were found to occur within its functional domain i.e., MH2 domain (323‒552 AA) of *SMAD4* gene (Fig. 6). Therefore, the present observation has added remarkable evidence to this study for putative NSHL target identification.

## Discussion

Heterogeneity in NSHL is still under the puzzled cube which has encouraged researchers to understand its accurate genetics. In the present study, authors have attempted to discover potential NSHL target genes and their functional variants using a computational approach. Initial prediction has identified the involvement of 23 genes in the inner ear development and hearing impairment pathways such as Notch signaling pathway, Wnt signaling pathway, gap junction pathway, adherens junction pathway, tight junction pathway, and JAK-STAT pathway. As per the literature evidence, all of these six pathways are appeared to have a crucial role in the NSHL progression. Several studies have been reported about the differential expression pattern of different functional genes in Notch signaling pathway which has multiple roles in the development of inner ear including regulation of hair cell, determination of neurons, sensory regions, and non-sensory regions [31]. It is also disclosed that, Wnt signaling pathway has a dominant role in the dorsal structure formation of the inner ear [32,33]. In addition, a critical role in hearing is directed through gap junction pathway and mutations in connected genes have been reported to cause a high incidence of human deafness [34]. Similarly, several physiological processes such as cochlear development, growth of auditory neurons, immune mediation, and planar cell alignment are maintained through Adherens junction pathway [35]. Again, in the inner ear, tight junction is important for the separation of endolymphatic and perilymphatic space to maintain the concentration gradient between endo and perilymph and the endocochlear potential [35,36]. Mutations in genes and/or proteins associated with this pathway are reported to cause hereditary HL [35,37]. Likewise, JAK-STAT signaling pathway balances the noise-induced damage to cochlear tissue and loss of hearing sensitivity. The imbalanced expression of STAT3 is caused by loud sound which leads to cochlear tissue damage and loss of hearing sensitivity [38]. All of this evidence stood in support of 23 genes as potential NSHL targets identified from the present study.

Afterward, participation of 16 genes (out of 23) in different biological processes such as inner ear development, homeostatic process, chemical homeostasis, signal transduction, inner ear auditory receptor cell differentiation, and regulation of response to external stimulus related to hearing impairment and NSHL progression was elucidated through study of their PPI network [39-41]. Therefore, all of these biological processes are providing the major key points to consider the involved genes as potential NSHL targets. The predicted strong functional association between these genes also supported the above hypothesis. From subsequent analysis, a strong regulatory interaction between two important NSHL target genes (*SMAD4* and *SNAI2*) with three established miRNAs (miR-183, miR-182, and miR-96) was obtained which have added a worthy value to this current study. The expressions of these three miRNAs in the human inner ear and their involvement in NSHL progression [15-18] have been studied well. According to the previous findings, both of these miRNAs (miR-182 and miR-183) down-regulate the *SMAD4* expression in human bladder, prostate cancer, and ovarian cancer [42-44]. Apart from these several others such as miR-183 regulates *ATP2B4, BTG1, EZRIN, GNG5, KCNJ14, NCS1, NEFL, PEX19, PPP2CA, SLC6A6, TREK-1, ZCCHC3* [18] and miR-182 regulates *CaV1.2, MITF, RDX *[15,18]. Similarly, miR-96 is known to regulate several genes including *MYRIP, AQP5, CaBP1, CaV1, CELSR1, CELSR2, GRID1, KCC2, MITF, RDX, RYK*, and *TFCP2L3* [17,18]. At the same time, a study in the mice model has confirmed the regulation of *SNAI2* gene by miR-96 [45].

As, the regulation of *SMAD4* and *SNAI2* through miR-183, miR-182, and miR-96 in human NSHL is still not clear, further investigation may provide proper direction into it.

Here, total of 13 deleterious nsSNPs (G352E, G352R, R361H, R361L, C363R, G386D, G395V, G491V, W509R, G510V, L533P, L533R, and L533V) were obtained within the functional region i.e., MH2 domain of the *SMAD4* gene. *SMAD4* is a multifunctional modulator, which regulates cell proliferation, differentiation, autoimmunity, pluripotency, plasticity, cell growth, apoptosis, autophagy, invasion, and metastasis. Deregulation of *SMAD4* is also associated with embryonic developmental diseases [46]. It has a vital role in the activation of Wnt signaling pathway [47]. The *SMAD4* variants G352E [48], G352R [49], R361H, R361L [48], C363R [50], G386D [49], W509R, G510V [50], L533P, L533R [48], and L533V [51] are previously identified in juvenile polyposis/hereditary hemorrhagic telangiectasia syndrome, whereas G491V is reported in colorectal cancer [51]. The appearance of most of these deleterious mutations in the MH2 domain of *SMAD4* protein has emphasized their importance for further study. MH2 domain is a multifunctional region that mediates differential interaction with other proteins, transcription factors, co-activators, and co-repressors. These interactions provide specificity and selectivity to *SMAD4* functions [52,53].

Interestingly, the association of a deleterious variant i.e., rs377767367 (G491V) in NSHL target *SMAD4* was discovered and is not reported in the NCBI ClinVar database for any disease conditions. Thus, present findings will not only provide insights into the genetics of NSHL but also help in further genetic counselling of NSHL after subsequent investigation.

NSHL is a critical hereditary disorder. Complexity in disease diagnosis increases due to its clinical and genetic heterogeneity. The present study has reported *SMAD4* as the most potential target gene in the HL development pathways. The discovery of important miRNAs, miR-183, and miR-182 in associated with the regulation of *SMAD4* has also supported the above hypothesis. Identification of pathogenic and deleterious nsSNPs within its functional domain is also added an additional clue to this study. Outcomes of the present investigation may be useful in understanding the genetics of NSHL and also throw light on the diagnosis modalities for NSHL. All of these findings are needed to experiment with for further validation.

## Figures and Tables

**Table 1.** Associated genes in signaling pathways of non-syndromic hearing loss development

Pathway	Pathway IDs	NSHL target genes	No. of genes
Notch signaling pathway	hsa04330	*NOTCH1, RBPJ*	2
Wnt signaling	hsa04310	*LRP5, SMAD4*	2
Gap junction	hsa04540	*RAF1, ADCY1, GJA1*	3
Adherens junction	hsa04520	*SMAD4, SNAI2, ACTB, ACTG1, FGFR1, MET*	6
Tight junction	hsa04530	*TJP2, ACTB, ACTG1, CLDN14, CLDN9, MARVELD2, MYH9, NEDD4, NF2, RDX*	10
JAK-STAT pathway	hsa04630	*IFNLR1, IL13, PTPN11, RAF1*	4

NSHL, non-syndromic hearing loss.

**Table 2.** NSHL targets involved in different biological processes obtained from STRING functional network analysis

Biological processes	GO IDs	NSHL associated gene
Inner ear development	GO:0048839	*NOTCH1, PTPN11*
Homeostatic process	GO:0042592	*RAF1, NOTCH1, MET, PTPN11, SMAD4*
Chemical homeostasis	GO:0048878	*RAF1, MET, PTPN11, SMAD4*
Signal transduction	GO:0007165	*RAF1, NOTCH1, MET, PTPN11, SMAD4, SNAI2, TJP2*
Regulation of response to external stimulus	GO:0032101	*RAF1, NOTCH1, MET, PTPN11, SNAI2*

NSHL, non-syndromic hearing loss.

**Table 3.** Identified pathogenic and deleterious nsSNPs in putative NSHL target SMAD4

*SMAD4* rsIDs	Mutation
rs377767345	G352E
rs121912581	G352R
rs377767347	R361H, R361L
rs377767348	C363R
rs121912580	G386D
rs377767367	G395V, G491V
rs377767369	W509R
rs377767371	G510V
rs377767382	L533P, L533R
rs377767381	L533V

nsSNP, non-synonymous single nucleotide polymorphism; NSHL, non-syndromic hearing loss.

## Supplementary Materials

Supplementary Fig. 1. Retrieved SNPs(rsIDs) of SMAD4 from dbSNP.

Supplementary Fig. 2. Screened deleterious rsIDs of SNAD4 through SIFT tool.

Supplementary Fig. 3. Deleterious rsIDs of SMAD4 identified through PredictSNP1.
