# Supplementary material for: Presentation of potential genes and deleterious variants associated with non-syndromic hearing loss: a computational approach
Source: Genomics Inform. 2022 Mar 31;20(1):e5. doi: 10.5808/gi.21070 (PMC9001992; doi:10.5808/gi.21070)
Supplement: Supplementary Fig. 1. — Retrieved SNPs(rsIDs) of SMAD4 from dbSNP. [file gi-21070suppl1.pdf]

Genes	SMAD4
**rsIDs from dbSNP**	rs7238500
	rs80338963
	rs80338964
	rs80338965
	rs121912576
	rs121912577
	rs121912578
	rs121912579
	rs121912580
	rs121912581
	rs138386557
	rs140743238
	rs149755320
	rs200772603
	rs281875320
	rs281875321
	rs281875322
	rs281875324
	rs377767323
	rs377767324
	rs377767325
	rs377767326
	rs377767327
	rs377767328
	rs377767329
	rs377767330
	rs377767331
	rs377767332
	rs377767333
	rs377767334
	rs377767335
	rs377767336
	rs377767338
	rs377767339
	rs377767340
	rs377767341
	rs377767342
	rs377767343
	rs377767344
	rs377767345
	rs377767346
	rs377767347
	rs377767348
	rs377767349
	rs377767350
	rs377767351
	rs377767352
	rs377767354
	rs377767355
	rs377767356
	rs377767357
	rs377767358
	rs377767359
	rs377767360
	rs377767361

rs377767362
rs377767363
rs377767364
rs377767365
rs377767366
rs377767367
rs377767368
rs377767369
rs377767370
rs377767371
rs377767372
rs377767373
rs377767374
rs377767375
rs377767376
rs377767377
rs377767378
rs377767379
rs377767380
rs377767381
rs377767382
rs377767383
rs377767384
rs377767385
rs377767386
rs377767387
rs397518413
rs483352871
rs587780124
rs587781359
rs587781618
rs587782209
rs587783060
rs727504151
rs730881952
rs730881954
rs730881956
rs730881957
rs746084369
rs757971589
rs773615487
rs779069779
rs786201200
rs786203560
rs786204125
rs786205514
rs863224400
rs863224507
rs864622252
rs869312781
rs876658694
rs876660079
rs876660150
rs876660556
rs876660720
rs878854765

rs878854769
rs956212866
rs1057519739
rs1057519740
rs1057519741
rs1057519962
rs1060500733
rs1060500734
rs1060500738
rs1060500739
rs1060500740
rs1060500742
rs1060500744
rs1064793271
rs1064795175
rs1064796471
rs1295343500
rs1316902116
rs1449334786
rs1555685142
rs1555685149
rs1555685156
rs1555685159
rs1555685248
rs1555685624
rs1555685925
rs1555685974
rs1555685978
rs1555686070
rs1555686071
rs1555686086
rs1555686469
rs1555686503
rs1555686506
rs1555686594
rs1555686600
rs1555686604
rs1555686608
rs1555686610
rs1555686616
rs1555686624
rs1555687377
rs1555687378
rs1555687386
rs1555687387
rs1555687388
rs1555687572
rs1555687599
rs1555687605
rs1568206107
rs1568206602
rs1568208715
rs1568211187
rs1568211588
rs1599181081
rs1599181251

rs1599182571
rs1599182586
rs1599182906
rs1599195400
rs1599195433
rs1599195489
rs1599196995
rs1599197105
rs1599204042
rs1599204121
rs1599204140

Supplementary Fig. 1. Retrieved SNPs(rsIDs) of SMAD4 from dbSNP.
