# Supplementary material for: Presentation of potential genes and deleterious variants associated with non-syndromic hearing loss: a computational approach
Source: Genomics Inform. 2022 Mar 31;20(1):e5. doi: 10.5808/gi.21070 (PMC9001992; doi:10.5808/gi.21070)
Supplement: Supplementary Fig. 2. — Screened deleterious rsIDs of SNAD4 through SIFT tool. [file gi-21070suppl2.pdf]

rs1599182571
rs1599182586
rs1599182906
rs1599195400
rs1599195433
rs1599195489
rs1599196995
rs1599197105
rs1599204042
rs1599204121
rs1599204140

**Genes**	**SMAD4**
**rsIDs from SIFT**	rs8

0338963 rs121912580
rs121912581 rs281875324
rs377767339 rs377767342
rs377767345 rs377767346
rs377767347 rs377767348
rs377767350 rs377767355
rs377767367 rs377767369
rs377767371 rs377767375
rs377767381 rs377767382

Supplementary Fig. 2. Screened deleterious rsIDs of SNAD4 through SIFT tool.
