# Supplementary material for: Presentation of potential genes and deleterious variants associated with non-syndromic hearing loss: a computational approach
Source: Genomics Inform. 2022 Mar 31;20(1):e5. doi: 10.5808/gi.21070 (PMC9001992; doi:10.5808/gi.21070)
Supplement: Supplementary Fig. 3. — Deleterious rsIDs of SMAD4 identified through PredictSNP1. [file gi-21070suppl3.pdf]

rs1599182571
rs1599182586
rs1599182906
rs1599195400
rs1599195433
rs1599195489
rs1599196995
rs1599197105
rs1599204042
rs1599204121
rs1599204140

**Genes**	**SMAD4**
**rsIDs from PredictSNP1**	rs377767339 rs281875324 rs377767342 rs377767345 rs121912581 rs80338963 rs377767347 rs377767348 rs377767350 rs377767355 rs121912580 rs377767367 rs377767369 rs377767371 rs377767375 rs377767382 rs377767381

Supplementary Fig. 3. Deleterious rsIDs of SMAD4 identified through PredictSNP1.
